# Bifurcation analysis informs Bayesian inference in the Hes1 feedback loop

**DOI:** 10.1186/1752-0509-3-12

**Published:** 2009-01-26

**Authors:** Catherine F Higham

**Affiliations:** 1 Faculty of Biomedical and Life Sciences, University of Glasgow, Glasgow, Scotland, UK

## Abstract

**Background:**

Ordinary differential equations (ODEs) are an important tool for describing the dynamics of biological systems. However, for ODE models to be useful, their parameters must first be calibrated. Parameter estimation, that is, finding parameter values given experimental data, is an inference problem that can be treated systematically through a Bayesian framework.

A Markov chain Monte Carlo approach can then be used to sample from the appropriate posterior probability distributions, provided that suitable prior distributions can be found for the unknown parameter values. Choosing these priors is therefore a vital first step in the inference process. We study here a negative feedback loop in gene regulation where an ODE incorporating a time delay has been proposed as a realistic model and where experimental data is available. Our aim is to show that *a priori *mathematical analysis can be exploited in the choice of priors.

**Results:**

By focussing on the onset of oscillatory behaviour through a Hopf Bifurcation, we derive a range of analytical expressions and constraints that link the model parameters to the observed dynamics of the system. Computational tests on both simulated and experimental data emphasise the usefulness of this analysis.

**Conclusion:**

Mathematical analysis not only gives insights into the possible dynamical behaviour of gene expression models, but can also be used to inform the choice of priors when parameters are inferred from experimental data in a Bayesian setting.

## Background

### Aims

Mathematical models can help biologists understand the mechanisms and dynamics behind their experimental observations (Tomlin et al. [1]). The most widely used approach to modelling the dynamics of a genetic network is to employ systems of ordinary differential equations (ODEs) (Voit [2] and de Jong [3]). These models have biological parameters, some of which can be measured experimentally and some of which cannot. Parameter estimation, that is, recovering unknown parameters from experimental data, is an important step towards obtaining a good model that can not only explain observed results but can also be used for prediction and "what if" scenarios.

The inference of parameters, from real biological data, within a Bayesian framework is a relatively new, albeit currently very active area. Bayesian inference and Markov chain Monte Carlo (MCMC) methods have been recently advocated for the estimation of model parameters from biological systems described by ODEs (Rogers et al. [4]). This paper aims to show that a general technique, using dynamical systems analysis to inform the choice of priors, can add value. The negative feedback loop studied here is a key feature of many complex biological networks and is referred to as a motif by Alon [5]. Hence our work will be of interest whenever larger networks are dealt with by a modular 'divide and conquer' approach. A Bayesian setting links the quantity that we are interested in, the probability that our parameters take certain values given the data, to two quantities that we can assign, the probability that we would have observed the measured data if the parameters took those values and our prior biological knowledge or ignorance about these parameters (Sivia [6]). Whereas traditional parameter estimation methods are deterministic and point valued (for example, COPASI [7]), these new methods use Bayes' Theorem to assign probabilities to parameter values and can handle noise inherently.

Using a Bayesian approach to parameter estimation for nonlinear dynamical systems throws up several challenges, and these typically increase when time delays are included. Key issues are

• dimensionality: models may involve several undetermined parameters,

• identification: different parameter combinations may produce similar dynamics, for example, increasing a production rate may be almost equivalent to decreasing a decay rate,

• local maxima: the likelihood function may have many locally optimal values.

MCMC methods [8] can go some way to addressing these difficult issues. In this work we show that *a priori *mathematical analysis can also be an effective tool. Our focus is on oscillatory time series, and we use the standard and widely applicable tools of Hopf bifurcation analysis and Lindstedt's method [9] in order to inform the choice of priors, thereby simplifying and focussing the parameter estimation process in order to find a particular parameter regime. More precisely, we follow the approach of Verdugo and Rand [10] in studying the case where oscillations arise via bifurcation through the delay parameter. Verdugo and Rand show that this leads to biologically realistic parameter values. We show here that inference with real data further supports this viewpoint.

The setting for our work is the biologically important instance where the expression of a gene is down regulated by its protein product. This arises, for example, with the p53 tumor suppressor protein whose intracellular activity is regulated through a feedback loop involving its transcriptional target [11]. We focus here on the case of the delayed Hes1 feedback loop featuring hes1 mRNA and Hes1 protein, where both mathematical models and quantitative experimental data are available in [12] and [13].

#### Biological Data

It has been observed that mRNAs for Notch signalling molecules such as the bHLH factor Hes1 oscillate with 2-hour cycles during somite segmentation, although the molecular mechanism of such oscillations remains to be fully determined. Hirata et al. [13] investigated the oscillations of mRNAs for Notch signalling molecules by examining the time course of hes1 mRNA and its Hes1 protein in detail. They observed that a single serum treatment induced a 2-hour cycle oscillation of hes1 mRNA in a variety of cultured cells and that Hes1 protein also oscillated in a 2-hour cycle after the single serum treatment with a delay of about 15 minutes relative to the hes1 mRNA oscillation. The half lives of hes1 mRNA and Hes1 protein were measured and the proteases for Hes1 protein degradation were identified.

Hirata et al. confirmed experimentally that degradation of Hes1 protein is required for hes1 mRNA increase and that *de novo *production of the protein is required for reduction of hes1 mRNA. These facts together support the theory that Hes1 is an essential component of a two hour cycle clock. They showed that the same mechanism applies to hes1 mRNA oscillation in the presomitic mesoderm.

**Figure 1:**
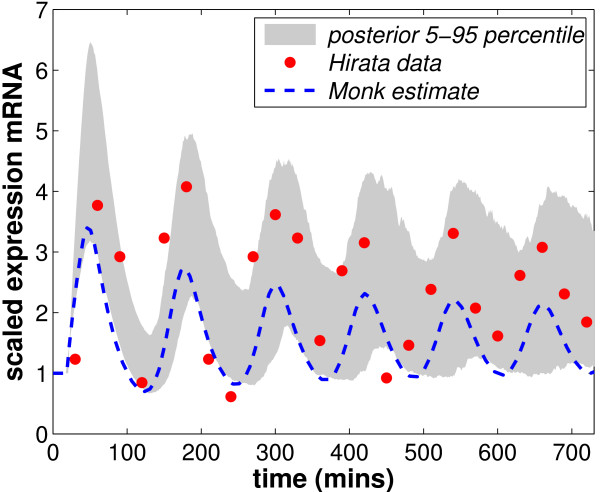
**Experiment 2: Hirata data comprises 25 mRNA values taken every 30 minutes over a 12 hour period**. Initial conditions are assumed to hold for 0 <*t *<*τ*.

The Hirata data comprises scaled hes1 mRNA expression levels every 30 minutes over a 12 hour period; see the discrete points in Figure 1.

A system featuring negative feedback and described by only two ODEs (one for each species, in this case hes1 mRNA and Hes1 protein), can be shown never to generate sustained oscillations [14]. Hirata et al. proposed that the observed oscillations could be modelled by introducing a Hes1 interacting factor as a third molecular species, but there is no experimental evidence for such an interacting factor. Monk [12] showed that observed oscillatory behaviour can be explained by incorporating a time delay.

#### Model for Hes1 Feedback Loop

Monk [12] suggests that the observed oscillations are due to the non-instantaneous nature of transcriptional and translational delays, and proposes a mathematical model where a delay is introduced to account for this feature. Monk's model was able to explain, via numerical simulations, the oscillation of hes1 mRNA and Hes1 protein in cultured cells observed by Hirata et al. Letting *m *(*t*) and *p *(*t*) denote the concentration of hes1 mRNA and Hes1 protein at time *t*, respectively, the model proposed by Monk for the Hes1 feedback loop takes the form

(1) $$\dot{m}=\frac{1}{1+{\left(p(t-\tau )/p_{0}\right)}^{n}}-\mu_{m}m,$$

(2) $$\dot{p}=m-\mu_{p}p,$$

where *μ*_*m *_and *μ*_*p *_are the rates of degradation of mRNA and protein, respectively, *p*_0 _is the normalised repression threshold, *n *is a hill coefficient and *τ *is a constant delay caused by transcription and translation. In this system, the Hes1 gene transcribes hes1 mRNA which passes from the nucleus to the cytoplasm. There it is translated into Hes1 protein. An unusual feature is that the Hes1 protein binds to the gene promoter and represses the transcription of hes1 mRNA. Following Monk, the equations have been rewritten so that the delay appears in the equation describing the regulation of transcription and not in the equation describing protein translation or synthesis. Throughout the rest of this paper, we will refer to *τ *as a transcriptional delay. The equations have also been rescaled so that the dynamics of the model are determined by five parameters. Using an ad hoc parameter fitting procedure, Monk showed that this model reproduced the broad features of the oscillation of hes1 mRNA and Hes1 protein in cultured cells observed by Hirata et al. [13].

Thus Monk [12] argues that the observed oscillatory behaviour is best accounted for by the introduction of a delay parameter that acts as a proxy for the many sub-processes that make up transcription and translation, rather than by introducing an unknown third agent [13]. By performing mathematical analysis of the model (1)–(2), we will show that the convincing but ad hoc parameter fitting exercise in [12] can be extended to a fully Bayesian setting.

#### Mathematical Analysis

Verdugo and Rand [10] gave some mathematical analysis of Monk's model [12] for the Hes1 feedback loop. In particular, they derived closed form approximations for the amplitude and frequency of oscillation, where oscillatory behaviour is assumed to arise through Hopf bifurcation in the delay parameter. The analysis in [10] applies to the case where the decay rates of hes1 mRNA and Hes1 protein, key components of the feedback, are equal, that is, *μ*_*m *_= *μ*_*p *_in (1)–(2). In this work, we study the more realistic case where the decay rates are allowed to be different, also focussing on oscillatory behaviour. By allowing these degradation rates to differ, we expose some interesting features of the nonlinear system under consideration; namely that oscillations of this type only occur when the difference between degradation rates is small. The novelty of this work lies in using bifurcation tools to aid what turns out to be a very difficult inference problem. Details of the mathematical analysis can be found in the methods section. Our results are of interest in their own right as a means to understand how the system dynamics are driven by the model parameters, but our main aim here is to inform the corresponding Bayesian inference problem.

### Experiments

In order to illustrate the use of mathematically informed priors when inferring model parameters we conducted two experiments. Experiment 1 uses synthetic data, computer generated from the mathematical equations describing the Monk model with known parameters. Experiment 2 looks at the published data of Hirata et al. Data is noisy and we have noted the error bars in the Hirata data when making assumptions about this type of extrinsic noise. Bayesian methods are very useful when dealing with this type of experimental noise because we can include noise as a hyper parameter, treat it as a nuisance parameter and integrate it out. Intrinsic noise will also come into play at the molecular level [15] and there is recent work on the subject of inferring parameters in stochastic models [16]. However, we do not believe that there is enough data to make it feasible to fit a stochastic model that would take into account intrinsic noise. We therefore use the ODE-based model in line with other authors [12] and [15]. Consequently, we are dealing with extrinsic and experimental noise. To make the synthetic data in Experiment 1 more realistic, Gaussian noise is added, based on the absolute size of the error bars of Hirata with a mean of zero and a standard deviation of 0.2. In our example, we have used larger initial conditions so the relative noise is lower. However, investigations with a higher level of noise to match the relative values from Hirata give broadly similar conclusions. The justification for adding Gaussian noise to make the data more realistic lies with the *Central Limit Theorem*, which shows that under appropriate conditions the overall effect of extrinsic noise sources will be normal [4]. An advantage of using synthetic data is that the parameters to be inferred are known and so the accuracy of the method to infer them can be evaluated. For Experiment 2, two of the parameters values, *μ*_*m *_and *μ*_*p*_, have been measured independently. So mathematically informed priors are complemented with biological priors where available.

In Experiment 2 (Hirata Data) there is data for both species *m *and *p *and so in Experiment 1 (Synthetic Data) we generate data for both species *m *and *p *as well. We will show that some aspects of the data for *p *inform the mathematical analysis, however the actual inference of parameters for both *m *and *p *is based on the data for *m *only. This is because protein expression levels are harder to measure accurately in practice so it is useful to establish what can be inferred from the more accurately measured species alone. With the Hirata data, there is also the added complication that the protein levels measured have been scaled in an undisclosed manner [15].

The location in time of the sampling points is clearly an important issue. For species that oscillate, it may be difficult to know in advance which time points will provide most information. In this work the real data is already available and hence our initial test was chosen to match those details. Generally, for oscillatory behaviour, we would expect that the key constraint on the sample times is that the sampling frequency should be sufficiently large.

### Methodology

We have some data and a proposed model. We use a Bayesian framework to infer the parameters underlying the model. In a Bayesian framework, the posterior pdfs are given by the prior pdfs modified by the likelihood of the data given those parameters. More precisely the posterior pdfs are proportional to the product of the likelihood and the priors. The posterior pdf for a particular parameter depends on the values of all the other parameters and are shown for individual parameters by integrating over the other parameter values. Hence the posterior pdfs that we show are marginal distributions for each parameter and not distributions for fixed values of the remaining parameters. The resulting equations for the posterior pdfs are intractable so we use MCMC methods to sample from the posterior distributions. This section spells out further details of this methodology which differs slightly for Experiment 1 and Experiment 2.

#### Bayesian Inference

Bayesian methods have several advantages over other approaches to parameter estimation [17]: use of background information, the ability to include uncertainity in all parameter values and the ease of making inferences about some parameters irrespective of the values of others. The development of high-speed computing means that the potential of Bayesian methods is being realised, but there are still many computational difficulties. In this section, we summarise the key features of Bayesian inference and highlight the technical decisions which have to be made. For more information about Bayesian analysis we refer readers to [6] and reviews such as [17].

Our initial state of knowledge (or ignorance) about the model parameters is encapsulated in the *prior probability*. The priors are then modified by the experimental measurements through the *likelihood function *to give us the *posterior probability*, which represents our new state of knowledge about the value of the parameters. A likelihood function needs to be chosen and after using a normal distribution for Experiment 1, we chose to base the likelihood function for Experiment 2 on the chi-squared distribution. The chi-squared distribution, *χ*^2^, has longer tails than the normal distribution and so encourages the inclusion of outliers in the data, which is important for the estimation of model parameters which influence in particular the amplitude of the oscillations. This proved to be an important consideration in Experiment 2, possibly because of experimental noise levels.

For Experiment 1, we therefore suppose that the probability of the *k*th datum having a value *x*_*k *_is given by *N *(*f*_*k*_, *σ*) where *f*_*k *_= *f*(*θ*, *t*_*k*_) is the true value of the function of parameters *θ *= {*p*_0_, *n*, *μ*_*m*_, *μ*_*p*_, *τ*} of interest, and the variance, *σ*, accounts for the error in its measurement. In our case, *f*_*k *_is the mRNA level *m *(*p*_0_, *n*, *μ*_*m*_, *μ*_*p*_, *τ*) at time *t*_*k *_from the delay differential equation model (1)–(2).

For Experiment 2, we suppose that the probability of the *k*th datum having a value *x*_*k *_is given by *χ*^2 ^(*f*_*k*_, *v*) where the degree of freedom *v*, accounts for the error in its measurement. Given that the Hirata data has been scaled, it will also be necessary to infer an additional scaling parameter, which we refer to as *k*_*s*_, so *θ *= {*p*_0_, *n*, *μ*_*m*_, *μ*_*p*_, *τ*, *k*_*s*_}.

For both experiments, we assume that the value of *σ *and *v *is known. Our inference about the value of *θ *is expressed, therefore, by the posterior pdf *p *(*θ *| {*x*_*k*_}, *I*) where *I *denotes all other background information; to help us calculate it, we use Bayes' Theorem [6]

(3) *p *(*θ *| {*xk*}, *v*, *I*) ∝ *p *({*x*_*k*_} | *θ*, *v*, *I*) × *p *(*θ*, *v*, *I*).

The relation in (3) is expressed using proportionality, because the term *p *(*data*|*I*) has been omitted from the denominator in the right hand side. This is fine for data analysis problems, such as this one, involving *parameter estimation*, since the missing denominator is simply a normalisation constant not depending explicitly on the parameters. However if we were to consider *model selection*, this term would play a crucial role, and is thus given the special name of *evidence*.

As in [4], we assume that the data are independent, so that the measurement of one datum does not influence what we can infer about the outcome of another (when given the values of *θ*). The *likelihood function *is then given by the product of the probabilities of obtaining the *N *individual data:

(4) $$p(\{x_{k}\}|\theta ,I)=\prod_{k=1}^{N}p(x_{k}|\theta ,I).$$

We assign a Gaussian pdf as prior for each parameter *θ*_*j*_, with mean *μ*_*j *_and variance *σ*_*j*_, where *j *= 1:5 in Experiment 1 and *j *= 1:6 in Experiment 2:

(5) $$p(\theta_{j}|\sigma ,I)=p(\theta_{j}|I)=\frac{1}{\sigma_{j}\sqrt{2\pi }}\exp\left(-\frac{{\left(\theta_{j}-\mu_{j}\right)}^{2}}{2\sigma_{j}^{2}}\right).$$

According to (3), if we multiply the prior stated in (5) by the likelihood function (4), we obtain the posterior pdf. Our approach here will be to deal with the posterior pdf numerically, using MCMC.

#### MCMC Method

MCMC techniques are used to sample from distributions and more generally to solve optimisation and integration problems in large dimensional spaces (Andrieu et al. [8]). Here, we use MCMC to sample from the posterior pdf defined in (3). The approach is, essentially, to compute candidate values for our unknown parameters by constructing a Markov chain with an appropriate invariant measure. The candidate values are chosen randomly using a *proposal function *and are either accepted or rejected depending on an *acceptance probability*. The frequency at which a particular parameter is successfully sampled is indicative of its *posterior probability*. Once we have enough samples from the solution space, they can be binned, smoothed and normalised to indicate the relative posterior likelihood of a given parameter. Sampling can start from any particular parameter value. The *proposal function *is chosen so that samples are drawn widely and performance can be monitored by the *acceptance rate*. With Markov chains, after a *burn in period*, the memory of the initial starting point is lost. At this point sampling will be exclusively from the required distribution. We need to establish at which point this has occurred. Gelman et al. [18] propose setting off parallel sampling chains and monitoring their convergence with a statistic $\hat{R}$. We have followed this convention. More details can be found in the Methods section and Tables 1 (Experiment 1) and 2 (Experiment 2).

**Table 1:** Experiment 1 (Synthetic Data).

*θ*	sp1	sp2	sp3	*σ*	acc rate	$\hat{R}$
*P*_0_	85	95	91	0.5	26	1.0009
*n*	4	5	5.3	0.7	34	1.0001
*μ*_*m*_	0.024	0.028	0.022	0.1	43	1.0004
*μ*_*p*_	0.036	0.034	0.033	0.1	33	1.0000
*τ*	19	19.2	18.7	0.01	20	1.0000

This table shows inputs and convergence information for 3 illustrative MCMC chains. For each parameter, θ we show the starting point (sp) of the Markov chains (columns 2–4), variance σ of the Gaussian proposal function (column 5), the acceptance rate (column 6) and statistic $\hat{R}$ (column 7).

**Table 2:** Experiment 2 (Hirata Data).

*θ*	sp1	sp2	sp3	*σ*	acc rate	$\hat{R}$
*P*_0_	99	103	103	0.55	29	1.0000
*n*	5	4.8	5.3	0.7	34	1.0003
*μ*_*m*_	0.028	0.03	0.03	0.3	28	1.0001
*μ*_*p*_	0.028	0.03	0.031	0.5	31	1.0002
*τ*	18	19	18.5	0.5	27	1.0001
*k*_*s*_	2.5	2.2	2	0.2	27	1.0000

This table shows inputs and convergence information for 3 illustrative MCMC chains. For each parameter, θ we show the starting point (sp) of the Markov chains (columns 2–4), variance σ of the Gaussian proposal function (column 5), the acceptance rate (column 6) and statistic $\hat{R}$ (column 7).

## Results and discussion

### Mathematical Analysis

We summarise the main analytical results here. Further details about the derivations of the formulae can be found in the Methods section.

Following [10] we can derive an expression for the equilibrium state of *m *and *p *(m* and p*, respectively, see equations (21)–(20)) of model (1)–(2) and show that by increasing the delay the system moves from a stable state to an unstable state. At a critical value *τ*_*cr *_of the time delay a Hopf Bifurcation occurs. For values of delay *τ *close to *τ*_*cr*_, the nonlinear system is expected to exhibit a periodic solution which can be expressed for both the mRNA and protein in terms of their amplitudes, *A *and *B*, respectively, their oscillatory frequency *ω *(at *τ*_*cr*_) and their phase difference *ϕ*. Algebraic manipulation of these expressions results in explicit formulae linking the model's biological parameters which generalise those in [10]. Specifically, at the Hopf bifurcation we have

(6) $$\tan\phi =\frac{\omega }{\mu_{p}},$$

(7) $$\frac{B}{A}=\sqrt{\omega^{2}+\mu_{p}^{2}},$$

(8) $$w^{2}=-\frac{\left(\mu_{m}^{2}+\mu_{p}^{2})\pm \sqrt{{\left(\mu_{m}^{2}+\mu_{p}^{2}\right)}^{2}-4(\mu_{m}^{2}}\mu_{p}^{2}-K^{2}\right)}{2}$$

(9) $$\tau_{cr}=\frac{1}{\omega }\arctan\left(\frac{\omega \mu_{p}+\omega \mu_{m}}{\omega^{2}-\mu_{m}\mu_{p}}\right),$$

(10) $$\sin\omega \tau_{cr}=\frac{\omega \mu_{p}+\omega \mu_{m}}{K},$$

(11) $$\cos\omega \tau_{cr}=\frac{\omega^{2}-\mu_{m}\mu_{p}}{K},$$

where

(12) $$K=\frac{n\beta }{p^{*}{\left(1+\beta \right)}^{2}},$$

and

(13) $$\beta ={\left(\frac{p^{*}}{p_{0}}\right)}^{n}.$$

The result (7) characterises the ratio between mRNA and protein amplitudes, but not their absolute values. To proceed we use Lindstedt's method which is a technique for uniformly approximating periodic solutions to ODEs when regular perturbation approaches fail. We regard the frequency as unknown in advance and solve by demanding that an appropriate series expansion contains no secular terms. The result is closed form approximate expressions for the amplitude and frequency of oscillation expressed in terms of the model parameters. For the derivation of these formulae and more details, see the Methods section. In summary, our approximation to *A*^2 ^and the observed frequency – may be written in the form

(14) *A*^2 ^= *f*_*A *_(*n*, *p*_0_, *μ*_*m*_, *μ*_*p*_) Δ,

(15) Ω = *ω *- *f*_Ω_(*n*, *p*_0_, *μ*_*m*_, *μ*_*p*_) Δ,

and substituting (14) into (7) gives an approximation for *B*^2^

(16) $$B^{2}=(\omega^{2}+\mu_{p}^{2})f_{A}(n,p_{0},\mu_{m},\mu_{p})\Delta ,$$

where *f*_*A *_and *f*_Ω _are positive functions defined in terms of the model parameters and Δ = *τ *- *τ*_*cr*_. As Δ increases we would expect *A *and *B *to increase proportionally to $\sqrt{\Delta }$ and Ω to decrease.

The frequency of oscillation, *ω*, takes a maximum that can be explicitly defined in terms of the model parameters. We may show that for any *μ*_*m*_*μ*_*p *_= *c*, a unique maximum value of *ω *occurs when *μ*_*m *_= *μ*_*p *_= $\sqrt{c}$. We may then find the value of *c *and hence *μ*_*m *_and *μ*_*p *_for which the maximum of *ω *occurs. We can use this information combined with equation (15) to deduce that *ω *> Ω. This result allows us to make an informed judgement about *ω *given Ω, which can be used in the setting of priors.

Biology meaningful solutions must also have *ω *> 0 and *τ*_*cr *_> 0. Imposing these conditions in equation (9) gives

(17) *ω*^2 ^> *μ*_*m*_*μ*_*p*_,

(18) *ωμ*_*m *_+ *ωμ*_*p *_<*K*,

from which we deduce that

(19) $$\mu_{m}\mu_{p}<\frac{1}{p_{0}{\left(\frac{2}{n-2}\right)}^{1/n}\left(\frac{n}{n-2}\right)}.$$

**Figure 2:**
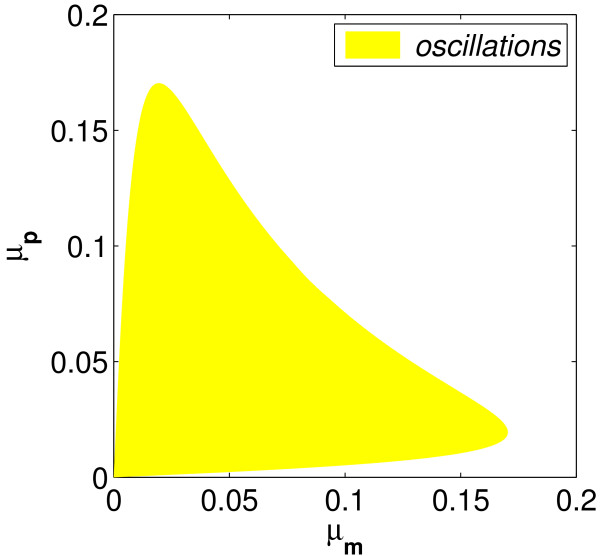
**Constrained region of the *μ*_*m*_-*μ*_*p *_plane where the inequality (19) predicts that oscillations may occur for arbitary *τ***. (In this picture *n *= 5 and *p*_0 _= 90).

This is a key result because we have defined a computable, finite region of the *μ*_*m *_- *μ*_*p *_plane in which oscillations can occur and in which we can search for oscillatory solutions; see Figure 2.

The data can tell us something about the parameters directly. Given sufficient data points from complete periodic cycles, the model parameter *μ*_*p *_can be approximated by the ratio of the average of *m *to the average of *p*. Further details can be found in the Methods section; see equation (70).

We now show how our results can be used to inform the choice of priors in our two inference experiments.

### Experiment 1 (synthetic data)

**Figure 3:**
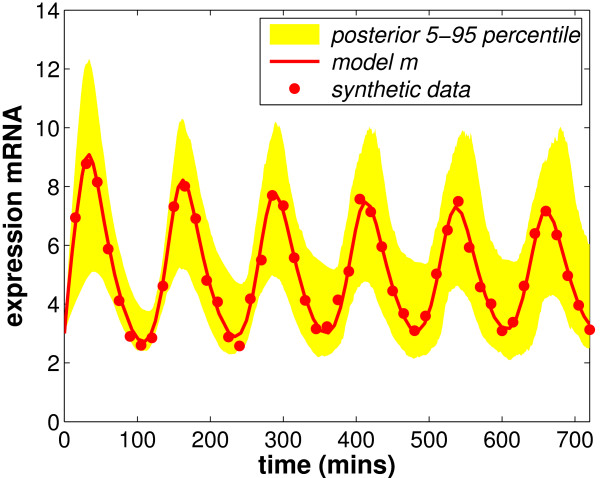
**Experiment 1: Synthetic data for the inference experiment comprising 49 mRNA values and 49 protein values (not shown here as they are not used directly in the inference problem)**. Data comes from the model with the following parameter values: *p*_0 _= 90, *n *= 5, *μ*_*m *_= 0.025, *μ*_*p *_= 0.035 and *τ *= 19.5. Initial conditions are 3 for the mRNA and 100 for the protein. Independent Gaussian noise (mean = 0 and standard deviation = 0.2) is then added to make the data more realistic.

**Figure 4:**
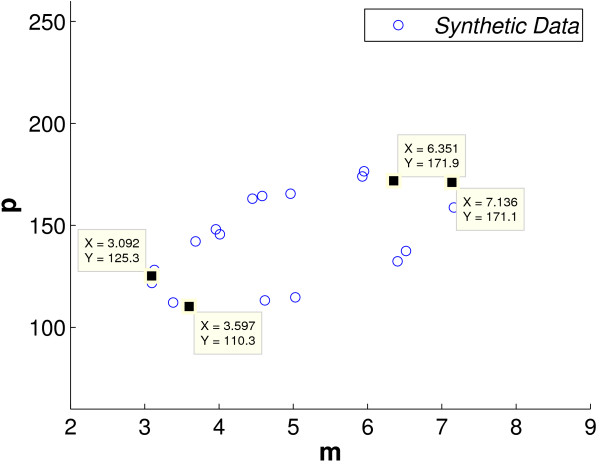
**Experiment 1: We use the observed data to estimate $\mu_{p}=\frac{m_{ave}}{p_{ave}}=\frac{4.9783}{144.8241}=0.0344$**. We use the data points representing *m*_*max *_= 7.136, *m*_*min *_= 3.092, *p*_*max *_= 171.9 and *p*_*min *_= 110.3 to calculate $\frac{B}{A}=\frac{7.136-3.092}{171.9-110.3}=0.0656$.

The synthetic data in Figure 3 comprises 49 mRNA values. Details of the parameter values used with the Monk equations and the added noise can be found in the caption of Figure 3. The corresponding 49 protein values were also generated and feature in Figure 4 where they are considered for the *μ*_*p *_prior. The protein values, as for Experiment 2, are not used in the inference problem. The following section describes how the mathematical analysis is used to find priors for the model parameters.

We can estimate *μ*_*p *_as $\frac{m_{ave}}{p_{ave}}$ from equation (70). In computing *m*_*ave *_and *p*_*ave*_, care needs to be taken to discard the transient data and to select data points from a complete number of cycles. In our example, as the oscillatory period appears to be about 2 hours, we take the last 20 data points which form approximately 5 cycles, see Figure 4. This gives an estimate for *μ*_*p *_of 0.0344. We estimate the ratio of the amplitudes $\frac{B}{A}$ using *B *≈ *m*_*max *_- *m*_*min *_and *A *≈ *p*_*max *_- *p*_*min*_, see Figure 4. Substituting *μ*_*p *_≈ 0.0344 and $\frac{B}{A}$ ≈ 0.0656 into (7) gives an estimate for *ω *of 0.0559. We now use the fact that *A*, *B *and *ω *are functions of the unknowns *p*_0_, *n *and *μ*_*m *_in order to solve numerically (7), (8), (14) and (15). This gives *p*_0 _≈ 90, *n *≈ 5 and *μ*_*p *_≈ 0.026. We also require a prior for *τ*. Lindstedt's method tells us that the closeness in value of *τ *and *τ*_*cr *_depends on the difference between *ω *and the observed frequency Ω. Our observed period is about 2 hours giving an observed frequency of 0.052. Substituting our estimates into (9) and (15) we obtain *τ*_*cr *_≈ 17.6567 and *τ *≈ 19.9508. See Table 3 for a summary of our priors for the synthetic experiment. The variances for the priors are based, in the case of *μ*_*m *_and *μ*_*p*_, on the decay rate measurements taken by Hirata et al., and in the case of the other variables on biological assumptions combined with the mathematical analysis.

**Table 3:** Experiment 1 (Synthetic Data).

*θ*	mean	std dev
*P*_0_	90	10
*n*	5	1
*μ*_*m*_	0.025	0.001
*μ*_*p*_	0.035	0.001
*τ*	18.5	1

This table details the Gaussian priors used for inferring from the synthetic data.

## Results

**Figure 5:**
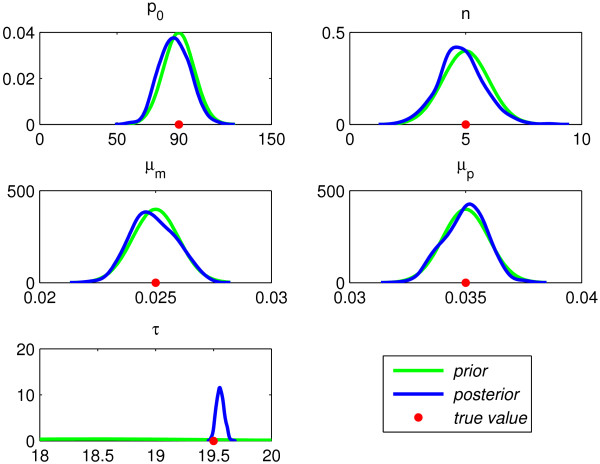
**Experiment 1: Prior pdfs, posterior pdfs and parameter value used in model**.

**Figure 6:**
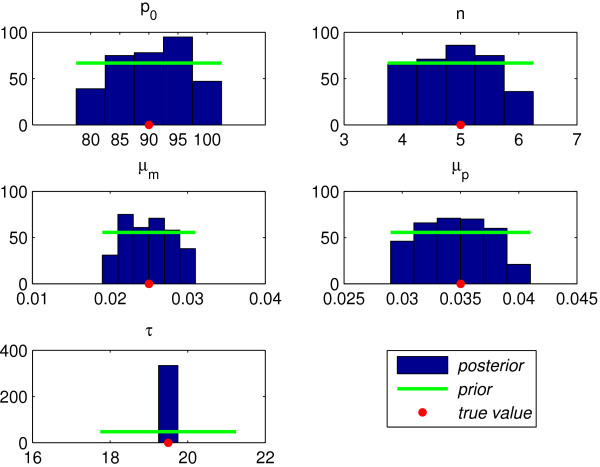
**Experiment 1: Binned posterior samples compared to mean ie uniform prior**.

Figure 5 shows the prior pdfs and the corresponding posterior pdfs for each of the parameters inferred in Experiment 1. The parameter values used in the model to simulate the data are shown on each x-axis with a dot. The peak of each curve indicates which parameter values are most probable, and a sharper peak indicates more certainty. For all parameters in Experiment 1 the peak lies over the input value to within visual accuracy. We are also interested in the extent to which the posterior pdfs, which express the probability of each parameter given the data, modify our prior beliefs. Here we used Gaussian priors with mean based on the point estimates outlined above and small variance. The posterior pdfs for *p*_0_, *n*, *μ*_*m *_and *μ*_*p *_provide only slight modifications of the prior pdfs suggesting that the *a priori *mathematical analysis has allowed us to select these priors very effectively. The fact that these mathematically informed priors, which are based on broad features of the data, are reasonably close to the posterior pdfs suggests that the bifurcation analysis is relevant and consistent with the data. The parameter *τ *shows the most change from prior to posterior. We can show from (16) that the amplitude of mRNA is sensitive to *τ*, and this experiment (and Experiment 2 below) suggests that the likelihood function is also strongly affected by *τ*. In Figure 6 we rerun the same experiment using uniform priors over the point estimates. We see more modification of the posterior pdfs, relative to the prior pdfs but, as shown by the flatness of the pdf curves, there is increased uncertainity in the prediction of parameter values. We ran several more experiments with different priors (not shown here) and conclude that when we apply more general priors we run into trouble with this hard inference problem and get stuck in suboptimal posteriors. This highlights the importance of exploiting whatever information can be gleamed from the structure of the model.

MCMC provides samples and in order to recover the posterior pdfs these samples are binned, smoothed and normalised. We can also simulate the dynamic activity associated with each of these samples using the associated parameter values. The variance of this dynamic activity at each time point indicates the likely range of activity that could have produced the data. The shaded area in Figure 3 shows this range (5–95 percentile) for Experiment 1. The fit of this area in terms of position and width shows how well we have recovered the model parameters from the data.

### Experiment 2 (biological data)

Hirata et al. measured the half lives of hes1 mRNA and Hes1 protein and estimated that hes1 mRNA is degraded with a half life of 24.1 ± 1.7 minutes and that Hes1 protein is degraded with a half life of 22.3 ± 3.1 minutes. These half lifes are close but not identical. Expressed as decay rates, the rate for hes1 mRNA is 0.0288 ± 0.002 and the rate for Hes1 protein is 0.0311 ± 0.004. We used these rates to give biologically informed priors for the parameters *μ*_*m *_and *μ*_*p*_. Using Lindstedt's method we obtained an expression for the observed oscillatory frequency Ω from equation (15). As *k*_2_Δ < 0 we have *ω *> Ω. In other words, we have a lower bound for *ω*. Hirata et al. observed an oscillatory period of around 2 hours which corresponds to an oscillatory frequency (in mins) of $\frac{2\pi }{120}$. Using this observation, we can argue that *ω *> 0.0524. Substituting *ω *= 0.0524, *μ*_*m *_= 0.0288 and *μ*_*p *_= 0.0311 into equation (8) gives *K *> 0.036. The value of *K *in equation (12) can be shown, via numerical computation, to be sensitive to the value of *n*, whereas the values of m* and p* are more sensitive to *p*_0 _than *n*. Given m* (or p* as m* = *μ*_*p*_p* from equation (21)) and the estimate for *K*, we can obtain point estimates from equations (21) and (12) of *n *≈ 5 and *p*_0 _≈ 100. We also require a prior for *τ*. Substituting our estimates for *ω*, *μ*_*m *_and *μ*_*p *_into equation (9) gives *τ*_*cr *_< 19.8 and we use this estimate of *τ*_*cr*_, which is close to *τ*, for choosing our prior for *τ*. We also need a scaling factor *k*_*s *_because the data has been scaled by a undisclosed constant, and this will also affect the initial condition. We set *k*_*s *_between 2 and 3, the initial condition of *p *to be 100 and *m *to be *k*_*s*_. See Table 4 for a summary of the priors.

**Table 4:** Experiment 2 (Hirata Data).

*θ*	mean	std dev
*P*_0_	100	10
*n*	5	1
*μ*_*m*_	0.0288	0.002
*μ*_*p*_	0.0311	0.004
*τ*	19	1
*k*_*s*_	2.2	0.1

This table details the Gaussian priors used for inferring the Hirata data. The priors for *μ*_*m *_and *μ*_*p *_are based on biological measurements.

## Results

**Figure 7:**
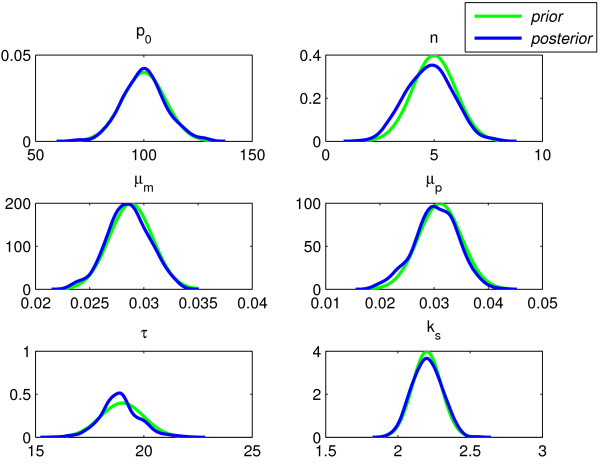
**Experiment 2: Prior and posterior pdfs**.

Figure 7 shows the prior pdfs and the corresponding posterior pdfs for each of the parameters inferred in Experiment 2. Once again, the customised priors are very close to the final pdfs. In this experiment, we chose a *χ*^2 ^distribution rather than a normal distribution for the likelihood, in order to encourage inclusion of any outlying data points.

The shaded area in Figure 1 shows the fit of the parameter estimates within the 5–95 percentile for Experiment 2. The early data points are covered by the prediction. Compared to Monk's results, there is broad agreement, however we predict a slightly longer delay (around 18.9 compared to Monk's 18.5) and slightly different values for *μ*_*m *_and *μ*_*p *_(around 0.0285 and 0.0305, respectively compared to Monk's 0.03 for both). We also inferred a different scaling factor which contributed significantly to the improved match to the Hirata et al. data that can be seen in Figure 1.

## Conclusion

We have shown that mathematical analysis can give insights into the possible dynamical behaviour of gene expression models. We derived a range of analytical expressions and constraints that link the model parameters to the observed dynamics of the system. The basis of the analysis is the assumption that oscillatory behaviour arises through Hopf bifurcation in the delay parameter. It is then possible to say for which range of parameters and to what extent this behaviour will occur. For the problem of inferring parameter values from data, we can use this analysis to inform the choice of priors. For these types of problems, where different parameter values can result in similar activity, identification of the parameters through the data can be ambiguous. MCMC methods can go some way towards resolving these difficulties but we showed here that mathematically informed priors can add value too. On the experimental data from Hirata et al., a full Bayesian MCMC computation improved on the parameter fit published by Monk, predicting non-equal values for mRNA and protein decay rates and a longer time delay for transcription.

## Methods

We describe here in more detail the mathematical and computational aspects of this work. We generalise the approach of Verdugo and Rand to the more biologically realistic case where the degradation rates of mRNA and protein are not assumed to be equal. The analysis reveals that we would expect to see oscillations when the difference between *μ*_*m *_and *μ*_*p *_is small which justifies, generally, the assumption that the degradation rates are almost equal.

### Stability of Equilibrium

Following the approach of Verdugo and Rand [10], equilibrium points for the system (1) and (2) are found by setting $\dot{m}$ = 0 and $\dot{p}$ = 0. After elimination and substitution, we obtain two expressions for p* and m*:

(20) $$\begin{array}{rrr} {\left(p^{*}\right)}^{n+1}+p_{0}^{n}p^{*}-\frac{p_{0}^{n}}{\mu_{m}\mu_{p}} & = & 0, \end{array}$$

(21) $$m^{*}=\mu_{p}p^{*}.$$

To find out whether m* and p* are stable, we linearize about these points and define *ζ *and *η *to be deviations from the equilibrium: *ζ *(*t*) = *m *(*t*) - m*, *η *(*t*) = *p *(*t*) - p*, and *η*_*d *_= *η *(*t *- *τ*). This results in the linear system:

(22) $$\dot{\zeta }=-\mu_{m}\zeta -K\eta_{d},$$

(23) $$\dot{\eta }=\zeta -\mu_{p}\eta ,$$

where *K *and *β *are given in (12) and (13). Equations (22) and (23) can be written as the second order DDE:

(24) $$\ddot{\eta }+(\mu_{m}+\mu_{p})\dot{\eta }+\mu_{m}\mu_{p}\eta +K\eta_{d}=0.$$

We now look for a solution of the form *η *= *e*^*λt*^, which leads to

(25) *λ*^2 ^+ (*μ*_*m *_+ *μ*_*p*_) *λ *+ *μ*_*m*_*μ*_*p *_+ *Ke*^-*λτ *^= 0.

First, we focus on the non-delay case, *τ *= 0. Here we have

(26) $$\lambda =\frac{-(\mu_{m}+\mu_{p})\pm \sqrt{{\left(\mu_{m}+\mu_{p}\right)}^{2}-4(\mu_{m}\mu_{p}+K)}}{2}.$$

Examination of equation (26) reveals that since *μ*_*m *_+ *μ*_*p *_> 0 and $\sqrt{{\left(\mu_{m}+\mu_{p}\right)}^{2}-4(\mu_{m}\mu_{p}+K)}<\mu_{m}+\mu_{p}$, the real part of *λ *will always be negative, implying stability. For

(27) 4 (*μ*_*m*_*μ*_*p *_+ *K*) > (*μ*_*m *_+ *μ*_*p*_)^2^

*λ *has a non-zero imaginary part as well as a negative real part and the equilibrium point (m*, p*) will be a stable spiral. When *μ*_*m *_= *μ*_*p*_, the inequality (27) becomes *K *> 0 which always holds. However when *μ*_*m *_≠ *μ*_*p *_this inequality becomes 4*K *> (*μ*_*m *_- *μ*_*p*_)^2^. In other words, we only have a stable spiral when |*μ*_*m *_- *μ*_*p*_| is sufficiently small. For larger values of |*μ*_*m *_- *μ*_*p*_| we will have two negative real roots for *λ*, resulting in a fixed stable point with no oscillations. We conclude that, overall, the non-delay model always has a linearly stable fixed point.

Following the approach in [10], we now assume, by continuity, that as *τ *increases the roots *λ *will cross the imaginary axis (this will not be at the origin because *μ*_*m *_> 0 and *μ*_*p *_> 0 and therefore the real and imaginary parts of *λ *cannot both be zero) at some critical value *τ *= *τ*_*cr*_, and for *τ *> *τ*_*cr *_the steady state (m*, p*) will lose stability giving rise to a Hopf bifurcation. We thus assume that for *τ *= *τ*_*cr *_the system (22)–(23) will exhibit a pair of pure imaginary eigenvalues ± *ω i *corresponding to the solution

(28) *ζ*(*t*) = *B *cos (*ωt *+ *ϕ*),

(29) *η*(*t*) = *A *cos *ωt*,

where *A *and *B *are the amplitudes of the *η *(*t*) and *ζ *(*t*) oscillations, and where *ϕ *is a phase angle. More generally, for values of delay *τ *close to *τ*_*cr*_, the nonlinear system is expected to relax to a periodic solution which can be written in the approximate form of equations (28) and (29).

Substituting equations (28) and (29) into equations (22) and (23) and matching time dependent terms results in the explicit formulae (6)–(11), which generalise those in [10].

### Lindstedt's method

Changing the first order system (22)–(23) into a second order DDE results in

(30) $$\ddot{\eta }+(\mu_{m}+\mu_{p})\dot{\eta }+\mu_{m}\mu_{p}\eta =-K\eta_{d}+H_{2}\eta_{d}^{2}+H_{3}\eta_{d}^{3}+\ldots$$

where the coefficients *K*, *H*_2 _and *H*_3 _are obtained by Taylor series expansion of the nonlinear term.

Assuming that the true solution is periodic, our goal is to find an analytical approximation that is valid for all *t*. The key idea is to regard the frequency *ω *as unknown in advance, and to solve for it by demanding that *η *contains no secular terms. We introduce a small parameter ϵ, and let Δ = *ϵ*^2^*δ*, with

(31) *η *= *ϵu*,

(32) *τ *= *τ*_*cr *_+ Δ = *τ*_*cr *_+ *ϵ*^2^*δ*.

We then stretch time by replacing the independent variable *t *by *σ*, where

(33) *σ *= Ω*t*.

We then have

(34) $$\Omega^{2}\frac{d^{2}u}{d\sigma^{2}}+(\mu_{m}+\mu_{p})\Omega \frac{du}{d\sigma }+\mu_{m}\mu_{p}u=-Ku_{d}+\epsilon H_{2}u_{d}^{2}+\epsilon^{2}H_{3}u_{d}^{3}+\ldots ,$$

where *u*_*d *_= *u *(*σ *- Ω*τ*). Expanding Ω in a power series in ϵ, omitting the *O *(ϵ) term for convenience, since it turns out to be zero, we have

(35) Ω = *ω *+ *ϵ*^2^*k*_2 _+ ...

Next we expand the delay term *u*_*d*_

(36) *u*_*d *_= *u *(*σ *- *ωτ*_*cr*_) - *ϵ*^2 ^(*k*_2 _*τ*_*cr *_+ *ωδ*) *u*' (*σ *- *ωτ*_*cr*_) + *O*(*ϵ*^3^)

Finally we expand *u *(*t*) in a power series in ϵ to obtain

(37) *u *(*σ*) = *u*_0 _(*σ*) + *ϵu*_1 _(*σ*) + *ϵ*^2^*u*_2 _(*σ*) + ...

Substituting and collecting terms we find

(38) $$\omega^{2}\frac{d^{2}u_{0}}{d\sigma^{2}}+(\mu_{m}+\mu_{p})\omega \frac{du_{0}}{d\sigma }+\mu_{m}\mu_{p}u_{0}=-Ku_{0}(\sigma -\omega \tau_{cr})$$

(39) $$\omega^{2}\frac{d^{2}u_{1}}{d\sigma^{2}}+(\mu_{m}+\mu_{p})\omega \frac{du_{1}}{d\sigma }+\mu_{m}\mu_{p}u_{1}=-Ku_{1}(\sigma -\omega \tau_{cr})+H_{2}u_{0}^{2}(\sigma -\omega \tau_{cr})$$

(40) $$\begin{array}{lll} \omega^{2}\frac{d^{2}u_{2}}{d\sigma^{2}}+(\mu_{m}+\mu_{p})\omega \frac{du_{2}}{d\sigma }+\mu_{m}\mu_{p}u_{2} & = & -Ku_{2}(\sigma -\omega \tau_{cr})+H_{3}u_{0}^{3}(\sigma -\omega \tau_{cr})\\ & & +2H_{2}u_{0}(\tau -\omega \tau_{cr})u_{1}(\tau -\omega \tau_{cr})\\ & & +K(k_{2}\tau_{cr}+\omega \delta ){u^{\prime }}_{0}(\tau -\omega \tau_{cr}). \end{array}$$

We take the solution of the *u*_0 _and *u*_1 _equations to have the form

(41) $$u_{0}(\sigma )=\hat{A}\cos\sigma ,$$

(42) *u*_1_(*σ*) = *m*_1 _sin 2*σ *+ *m*_2 _sin 2*σ *+ *m*_3_,

where *A *= $\hat{A}\epsilon$ from (29) and (31). Next we substitute (41) and (42) into (40) to give

$$\begin{array}{cc} \begin{array}{r} \omega^{2}(-4m_{1}\sin2\sigma -4m_{2}\cos2\sigma )+\\ \omega (\mu_{m}+\mu_{p})(2m_{1}\cos2\sigma -2m_{2}\sin2\sigma )+\\ Km_{1}(\sin2\sigma \cos2\omega \tau_{cr}-\cos2\sigma \sin2\omega \tau_{cr})+\\ Km_{2}(\cos2\sigma \cos2\omega \tau_{cr}-\sin2\sigma \sin2\omega \tau_{cr})+\\ Km_{3}+\mu_{m}\mu_{p}(m_{1}\sin2\sigma +m_{2}\cos2\sigma ) \end{array} & \begin{array}{ll} = & \frac{1}{2}H_{2}{\hat{A}}^{2}\cos2\sigma \cos2\omega \tau_{cr}+\\ & \frac{1}{2}H_{2}{\hat{A}}^{2}\sin2\sigma \sin2\omega \tau_{cr}+\frac{1}{2}H_{2}{\hat{A}}^{2}. \end{array} \end{array}$$

Collecting sin 2*σ *terms gives

$$\begin{array}{cc} \begin{array}{r} -4\omega^{2}m_{1}-2(\mu_{m}+\mu_{p})\omega m_{2}+\\ Km_{1}\cos2\omega \tau_{cr}+Km_{2}\sin2\omega \tau_{cr}+\mu_{m}\mu_{p}m_{1} \end{array} & \begin{array}{ll} = & \frac{1}{2}H_{2}{\hat{A}}^{2}\sin2\omega \tau_{cr}. \end{array} \end{array}$$

Collecting cos 2*σ *terms gives

$$\begin{array}{cc} \begin{array}{r} -4\omega^{2}m_{2}-2(\mu_{m}+\mu_{p})\omega m_{1}+\\ Km_{1}-\sin2\omega \tau_{cr}+Km_{2}\cos2\omega \tau_{cr}+\mu_{m}\mu_{p}m_{2} \end{array} & \begin{array}{ll} = & \frac{1}{2}H_{2}{\hat{A}}^{2}\cos2\omega \tau_{cr}. \end{array} \end{array}$$

Collecting other terms gives

$$\begin{array}{lll} Km_{3}+\mu_{m}\mu_{p}m_{3} & & \\ & = & \frac{1}{2}H_{2}{\hat{A}}^{2}. \end{array}$$

Letting

(43) *α *= -4*ω*^2 ^+ *K *cos2*ωτ*_*cr *_+ *μ*_*m*_*μ*_*p*_,

(44) *β *= -2*ω*(*μ*_*m *_+ *μ*_*p*_) + *K *sin 2*ωτ*_*cr*_,

(45) $${\hat{H}}_{1}=\frac{1}{2}H_{2}{\hat{A}}^{2}\sin2\omega \tau_{cr},$$

(46) $${\hat{H}}_{2}=\frac{1}{2}H_{2}{\hat{A}}^{2}\cos2\omega \tau_{cr},$$

(47) $${\hat{H}}_{3}=\frac{1}{2}H_{2}{\hat{A}}^{2},$$

we find that

(48) $$m_{1}=\frac{\alpha {\hat{H}}_{1}-\beta {\hat{H}}_{2}}{\alpha^{2}+\beta^{2}},$$

(49) $$m_{2}=\frac{\alpha {\hat{H}}_{1}+\beta {\hat{H}}_{2}}{\alpha^{2}-\beta^{2}},$$

(50) $$m_{3}=\frac{{\hat{H}}_{3}}{K+\mu_{m}\mu_{p}}.$$

Now we substitute (41) and (42) into (38) and equate to zero the coefficients of the resonant terms sin *σ *and cos *σ*. This gives

$$\begin{array}{cc} \begin{array}{r} k_{2}(-\mu_{m}-\mu_{p}+K\tau_{cr}\cos\omega \tau_{cr})\\ +\omega \delta K\cos\omega \tau_{cr} \end{array} & \begin{array}{ll} = & \frac{3}{4}H_{3}{\hat{A}}^{2}\sin\omega \tau_{cr}+\\ & H_{2}^{2}{\hat{A}}^{2}(m_{1}\cos\omega \tau_{cr}+m_{2}\sin\omega \tau_{cr}+2m_{3}\sin\omega \tau_{cr}) \end{array} \end{array}$$

and

$$\begin{array}{cc} \begin{array}{r} k_{2}(-2\omega -K\tau_{cr}\sin\omega \tau_{cr})\\ -\omega \delta K\sin\omega \tau_{cr} \end{array} & \begin{array}{ll} = & \frac{3}{4}H_{3}{\hat{A}}^{2}\cos\omega \tau_{cr}+\\ & H_{2}^{2}{\hat{A}}^{2}(-m_{1}\sin\omega \tau_{cr}+m_{2}\cos\omega \tau_{cr}+2m_{3}\cos\omega \tau_{cr}). \end{array} \end{array}$$

Let

(51) *c*_1 _= -*μ*_*m *_- *μ*_*p *_+ *Kτ*_*cr *_cos*ωτ*_*cr*_,

(52) *c*_2 _= -2*ω *- *Kτ*_*cr *_sin *ωτ*_*cr*_,

(53) $$d_{1}=\frac{3}{4}H_{3}\sin\omega \tau_{cr}+H_{2}^{2}(m_{1}\cos\omega \tau_{cr}+m_{2}\sin\omega \tau_{cr}+2m_{3}\sin\omega \tau_{cr}),$$

(54) $$d_{2}=\frac{3}{4}H_{3}\cos\omega \tau_{cr}+H_{2}^{2}(-m_{1}\sin\omega \tau_{cr}+m_{2}\cos\omega \tau_{cr}+2m_{3}\cos\omega \tau_{cr}).$$

Then we have

(55) $$A^{2}=\frac{\left(c_{2}\cos\omega \tau_{cr}+c_{1}\sin\omega \tau_{cr}\right)}{d_{1}c_{2}-c_{1}d_{2}}\omega \Delta K,$$

(56) $$k_{2}=-\frac{d_{2}\cos\omega \tau_{cr}+d_{1}\sin\omega \tau_{cr}}{c_{1}d_{2}-c_{2}d_{1}}\omega \delta K,$$

Multiplying (56) by ϵ^2 ^and substituting back into (35) gives

(57) Ω = *ω *+ *k*_2_Δ,

which has the form of equation (15) (noting that *k*_2 _is negative).

### Maximum *ω*

We have seen that *K *takes the same value for any *μ*_*m*_*μ*_*p *_= *c *where *c *is a constant. We are interested in investigating *ω *along *μ*_*m*_*μ*_*p *_= *c*. We will show that *ω *has a unique maximum along this line and the maximum value of *ω *occurs when *μ*_*m *_= *μ*_*p *_= $\sqrt{c}$. Rearranging equation (8) gives

(58) $$\omega^{4}+(\mu_{m}^{2}+\mu_{p}^{2})\omega^{2}+\mu_{m}^{2}\mu_{p}^{2}-K^{2}=0.$$

Substituting *μ*_*m*_*μ*_*p *_= *c *and *K *= *c*_*K *_into the above equation gives:

(59) $$\omega^{4}+\omega^{2}\left(\frac{c}{\mu_{p}^{2}}+\mu_{p}^{2}\right)\omega^{2}+c^{2}-c_{K}^{2}=0.$$

Differentiating w.r.t. *μ*_*p *_gives:

(60) $$4\omega^{3}\frac{d\omega }{d\mu_{p}}+2\omega \frac{d\omega }{d\mu_{p}}\left(\frac{c^{2}}{\mu_{p}^{2}}+\mu_{p}^{2}\right)+\omega^{2}\left(-\frac{2c^{2}}{\mu_{p}^{3}}+2\mu_{p}\right)=0,$$

So $\frac{d\omega }{d\mu_{p}}$ = 0 when

(61) $$\begin{array}{r} \omega^{2}\left(\frac{-2c^{2}}{\mu_{p}^{3}}+2\mu_{p}\right)=0\\ \frac{-2c^{2}}{\mu_{p}^{3}}+2\mu_{p}=0 \end{array}$$

giving

(62) $$\mu =\sqrt{c}.$$

As *μ*_*m*_*μ*_*p *_= *c*, we have *μ*_*m *_= *μ*_*p *_= $\sqrt{c}$. The maximum value of *ω *therefore occurs when *μ*_*m *_= *μ*_*p *_and has the form

(63) $$\omega =\sqrt{K-\mu^{2}}.$$

Writing *K *- *μ*_2 _in terms of *β *gives

(64) $$K-\mu^{2}=\frac{n\beta -(1+\beta )}{p_{0}\beta^{1/n}{\left(1+\beta \right)}^{2}}.$$

Differentiating with respect to *β *gives

(65) $$\frac{\beta^{1/n}{\left(1+\beta \right)}^{2}(n-1)-(n\beta -\beta -1)\left(\frac{1}{n}\beta^{1/n-1}{\left(1+\beta \right)}^{2}+2\beta^{1/n}(1+\beta )\right)}{p_{0}{\left(\beta^{1/n}{\left(1+\beta \right)}^{2}\right)}^{2}}.$$

Setting this expression to zero and dividing by *β*^1/*n *^(1 + *β*)/*p*_0 _gives

(66) $$n+n\beta -\beta -1-(n-1)(1/n(1+\beta )+2\beta )+\frac{1}{n\beta }(1+\beta )+2=0,$$

which simplifies to

(67) (*n*^2 ^- 1) *β*^2 ^- (*n*^2 ^+ 2) *β *- 1 = 0.

### Can the data tell us anything about the parameters directly?

Integrating equation (2) gives

(68) $$\int_{0}^{T}\dot{p}(t)dt=\int_{0}^{T}m(t)dt-\mu_{p}\int_{0}^{T}p(t)dt.$$

From the Fundamental Theorem of Calculus, it follows that

(69) $$p(T)-p(0)=\int_{0}^{T}m(t)dt-\mu_{p}\int_{0}^{T}p(t)dt.$$

If *p *is periodic and *T *is a multiple of the period then the left-hand side of this equation vanishes and the right-hand side may be written *m*_*ave *_- *μ*_*p*_*p*_*ave*_. This gives us the result

(70) $$\mu_{p}=\frac{m_{ave}}{p_{ave}}.$$

This allows us, from appropriate data sets, to infer the parameter *μ*_*p *_by averaging data from *m *and *p*. This, of course, presupposes that the period *T *can be estimated accurately and that there is enough data to form good approximations to the averages.

We remark that *μ*_*m *_cannot be approximated this way because of the nonlinearity in (1).

### MCMC (Metropolis Hastings) Method

Our approach was to choose candidate values for our unknown parameters, $\theta_{j}^{*}$, (*j *= 1 ... 5 in Experiment 1 and *j *= 1 ... 6 in Experiment 2) in logspace, based on the current value of $\theta_{j}^{\left(i\right)}$, using a Gaussian *proposal distribution*, $q\left(\theta_{j}^{*}|\theta_{j}^{\left(i\right)}\right)$ given by

(71) $$q\left(\theta_{j}^{*}|\theta_{j}^{\left(i\right)}\right)=\frac{1}{\sigma_{j}\sqrt{2\pi }}\exp\left(-\frac{{\left(\theta_{j}^{*}-\theta_{j}^{\left(i\right)}\right)}^{2}}{2\sigma_{j}^{2}}\right).$$

*σ*_*j *_values for the proposal rates are chosen in order that the acceptance rates are between 20% and 35%.

The candidate values are accepted or rejected with an *acceptance probability*, A(*θ*^(*i*)^, θ *), given by

(72) $$A\left(\theta_{j}^{\left(i\right)},\theta_{j}^{*}\right)=\min\left\{1,\frac{\hat{p}\left(\theta_{j}^{*}\right)q\left(\theta_{j}^{\left(i\right)}|\theta_{j}^{*}\right)}{\hat{p}\left(\theta_{j}^{\left(i\right)}\right)q\left(\theta_{j}^{*}|\theta_{j}^{\left(i\right)}\right)}\right\},$$

where

(73) $$\hat{p}=p(D|\theta_{j})p(\theta_{j}),$$

(74) $$p(D|\theta_{j})=p(\{x_{k}\}|\theta_{j},\sigma ,I),$$

and

(75) $$p(\theta_{j})=N(\mu_{j},\sigma_{pj}).$$

A pseudo-code outline for the MCMC Metropolis Hastings algorithm is as follows:

**step 1 **Initialise $\theta_{j}^{\left(0\right)}$

**step 2 **For *i *= 0 to *N*:

Sample *r *~ N(0, 1)

Sample $\theta_{j}^{*}\sim q\left(\theta_{j}^{*}|\theta_{j}^{\left(i\right)}\right)$ (choosing *j *at random

If $r<A\left(\theta_{j}^{\left(i\right)},\theta_{j}^{*}\right)=\min\left\{1,\frac{\hat{p}\left(\theta_{j}^{*}\right)q\left(\theta_{j}^{\left(i\right)}|\theta_{j}^{*}\right)}{\hat{p}\left(\theta_{j}^{\left(i\right)}\right)q\left(\theta_{j}^{*}|\theta_{j}^{\left(i\right)}\right)}\right\}$:

$$\theta_{j}^{\left(0\right)}=\theta_{j}^{*}$$

else

$$\theta_{j}^{\left(0\right)}=\theta_{j}^{\left(i\right)}$$
